# Effect of New Surfactants on Biological Properties of Liquid Soaps

**DOI:** 10.3390/molecules27175425

**Published:** 2022-08-25

**Authors:** Emilia Klimaszewska, Daria Wieczorek, Sławomir Lewicki, Marta Stelmasiak, Marta Ogorzałek, Łukasz Szymański, Ryszard Tomasiuk, Leszek Markuszewski

**Affiliations:** 1 Department of Cosmetology, Faculty of Medical Sciences and Health Sciences, Kazimierz Pulaski University of Technology and Humanities in Radom, 26-600 Radom, Poland; 2 Department of Technology and Instrumental Analysis, Institute of Quality Science, Poznan University of Economics and Business, 61-875 Poznan, Poland; 3 Department of Medicine, Faculty of Medical Sciences and Health Sciences, Kazimierz Pulaski University of Technology and Humanities in Radom, 26-600 Radom, Poland; 4 Department of Molecular Biology, Institute of Genetics and Animal Biotechnology, Polish Academy of Sciences, 05-552 Jastrzębiec, Poland; 5 Department of Physicochemistry and Materials Technology, Faculty of Chemical Engineering and Commodity Science, Kazimierz Pulaski University of Technology and Humanities in Radom, 26-600 Radom, Poland

**Keywords:** liquid soaps, amphoteric surfactant, irritating effect, keratinocytes, zein number, cytotoxicity

## Abstract

Liquid soaps are the basic cosmetics used to clean the skin of the hands. Frequent hand washing prevents viral contamination but may damage the skin’s hydro-lipid layer, leading to various types of irritation. Therefore, four liquid soap formulas were developed with three amphoteric surfactants: Cocamidopropyl Betaine (LS II), CocamidopropylHydroxysultaine (LS III), and newly synthesized Evening PrimroseaamidopropylSulfobetaine (LS IV). We evaluated the skin irritating potential (zein number, bovine albumin test) and cytotoxicity (AlamarBlue™, Cell viability, and Cell cycle assays) on HaCaT cell line. We observed lower values of the zein number and bovine albumin tests after adding soaps with surfactants (the highest differences in LS IV) compared to the base soap (LS I). However, LS I and LS II did not differ in cytotoxic assays. Therefore, adding LS III and LS IV seems potentially more dangerous to the cells. However, it should be noted that cells were continuously exposed to liquid soaps for more than 24 h, so its cytotoxic effects after dermal use in humans may be unnoticeable. Concluding, results suggest that the newly synthesized LS IV should improve the safety of liquid hand washing soaps.

## 1. Introduction

Frequent hand washing is the simplest, cheapest, and most effective way to reduce infections. For this purpose, liquid soaps are numerous on the market and are most often used. These are cosmetic products designed to maintain proper hand hygiene. From a chemical point of view, they are not soaps, that is, salts of higher fatty acids formed in the reaction of hydrolytic saponification of triacylglycerols with hydroxides, but they are usually aqueous solutions of surfactants with various additives. Liquid soaps were introduced to the market in the 1980s. These products allow for hygienically dispensing the right amount without touching the entire product, which is the case with traditional bars. They also have a lower pH compared to traditional soaps [1]. Surfactants from the anionic, nonionic, and amphoteric groups are present in liquid soaps’ composition. In addition to surfactants, raw materials used in the production of liquid soaps are various types of plant extracts, pH regulators, rheology modifiers, dyes, fragrances, humectants, and lubricants. In recent years, liquid soap design in accordance with sustainable development deserves special attention. In addition, consumers pay special attention to the impact of such products on health and the environment. According to a study by Yao et al., respondents are willing to pay more for liquid soap with reduced environmental and health impacts [2].

The cosmetic market offers both regular and antiseptic liquid soaps. They contain anionic, nonionic, and amphoteric surfactants as well as additional substances such as humectants. Some liquid soaps can be based on soap, a salt of a long-chain carboxylic acid. It is usually potassium soap enriched with various additives: consistency agents, glycerine, or surfactants. The composition of antiseptic liquid soaps is based on surfactants but also antiseptic substances. Antiseptics used in the production of liquid soaps include iodine derivatives, mercury derivatives, zinc and copper salts, benzoic, lactic, and salicylic acids, and quaternary amines. These substances show bacteriostatic, cleansing, and disinfecting properties. In the literature, the effectiveness of antiseptic soaps compared to regular soaps is debatable. Some authors believe that the use of antimicrobial soap does not always provide significant inactivation of bacteria [3,4]. They also highlight the adverse effects of antiseptic actives on human health and the environment, such as antibiotic resistance, acute and chronic toxicity, endocrine disruption, allergies, and bioaccumulation.

For both regular and antiseptic liquid soaps, the safety of products concerning hand skin is essential. As a result of frequent washing, hand skin is exposed to prolonged exposure to water and other chemical or physical agents. This may induce a number of pathophysiological changes, such as disruption of the epidermal barrier, impairment of keratinocytes, release of pro-inflammatory cytokines, activation of the skin immune system, and delayed-type hypersensitivity reactions. The following adverse dermatological changes may occur: from the development of cutaneous xerosis (dry skin) to contact dermatitis (especially the irritant subtype) and even allergic contact dermatitis. [5]. It is worth noting that hygiene has also taken on a different meaning these days. The COVID-19 pandemic caused the use of hygiene products such as liquid soaps has become a daily routine, practiced even a dozen times a day to protect against the multiplication of this dangerous virus. WHO and UNICEF recommend frequent hand washing with soap under running water for at least 20 s as a primary measure to reduce the spread of the COVID-19 virus [6]. Among others, Jefferson’sgroup confirmed that hand washing reduces the transmission of respiratory viruses by 45–55% [7,8]. Furthermore, Przekwas and Chen, in their work, documented that frequent hand washing can prevent virus infection [9]. They also pointed out that continuous hand washing with soap and water contributes to damage to the skin’s hydro-lipid layer, which may lead to various types of irritation in consumers. This is also emphasized by the American Contact Dermatitis Society, which predicts an increase in both contact irritation and allergic contact dermatitis of the hands [10].

To prevent the harmful effects of cleansers, emulsion skin care products are most often recommended after hand washing. These products should be applied generously, several times a day, especially immediately after hand washing. To effectively improve the skin barrier, combining humectants with occlusive emollients in these products is most often recommended. Humectants (e.g., glycerine, urea, propylene glycol) have the ability to attract water from the environment and from deeper skin layers to the stratum corneum. On the other hand, emollients (e.g., lanolin, mineral and vegetable oils, waxes) act occlusively on the skin preventing water loss and soothing irritation. Oily creams and ointments are more often recommended to provide better protection against xerosis than light emulsions like lotions. Additionally, fragrance-free and hypoallergenic products are recommended to reduce the risk of contact sensitization [5]. Another way of alleviating the negative effects of washing agents is replacing the commonly used, also in liquid soaps, irritating anionic surfactants (e.g., Sodium Laureth Sulfate, Sodium Lauryl Sulfate) with compounds with a milder effect on the skin. Various ways of reducing the irritant effect caused by anionic surfactants are presented in the literature. Work has mainly focused on the introduction of “mild” surfactants into formulation [11,12,13,14], protein hydrolysates [15,16], hydrophobic substances [17], or polymers [18]. The mechanism of action of such additives is usually related to an increase in micelle size or the formation of appropriate complexes in order to reduce the concentration of free surfactant monomers in the solution. Therefore, the present study’s goal was to assess the effect of the addition of amphoteric surfactants: Cocamidopropyl Betaine, CocamidopropylHydroxysultaine, and a newly synthesized amphoteric from Evening Primrose oil (Evening PrimroseamidopropylSulfobe-taine) to the soap on the safeness of newly developed products.

## 2. Results

### 2.1. Irritating Properties

The test with bovine albumin is based on determining the degree of protein denaturation by measuring the pH of a bovine albumin solution in a solution of the tested washing agent. In this test, due to the denaturation of this protein with a chemical structure similar to keratin, the building protein of the epidermis, changes in the structure of albumin occur as a result of contact with surfactants, which translates into changes in the pH of the solution. The incorporation of anionic surfactants into the albumin solution binds them to the cationic groups of the albumin protein. In order to neutralize the negative charge of the protein resulting from the predominance of anionic groups in its molecule, the adsorption of protons from the solvent takes place. This leads to an increase in the pH of the solution. It should be noted that the higher the pH of the solution increases, the stronger the irritating effect of surfactants [19,20,21].

**Figure 1:**
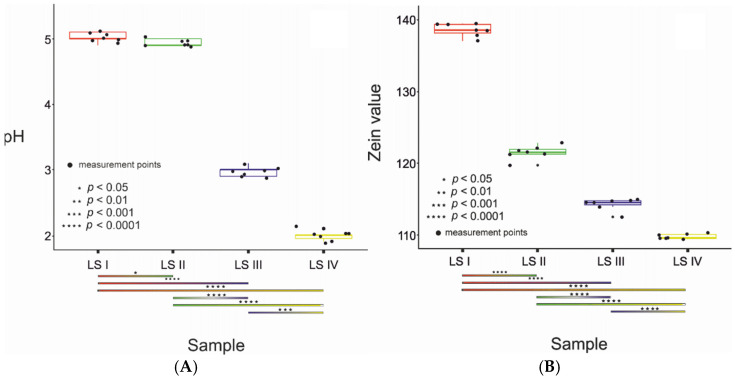
Bovine albumin assay (**A**) and zein assay (**B**) of tested soaps (LS I—base; in following soaps addition of 4% (*w/w*) surfactants: LS II—cocamidopropyl betaine; LS III—addition of cocamidopropyl hydroxysultaine; LS IV—addition of evening primroseamidopropyl sulfobetaine). Results were calculated from the formula: (end pH—initial pH)/initial pH, and presented as median with quartiles (25% and 75%), n = 7, three independent experiments.

The mean LS 1 (base) pH increase measured in the bovine albumin test was 5.77 after 48 h incubation, which after conversion, gave the result of 5.029 ± 0.069. The addition of LS II, LS III, and LS IV significantly decreased the assay values, with the highest differences observed LS IV (pH 5.609, after conversion: 1.986 ± 0.063, *p* < 0.001). Mean LS 1 (base) obtained in zein test was 138.9 ± 0.8 mg of nitrogen (N) in 100 mL of sample. The addition of tested ingredients significantly reduced the amount of nitrogen. It amounted to: 121.9 ± 0.9 (LS II, *p* < 0.001), 114.7 ± 0.8 (LS III, *p* < 0.001) and 110.2 ± 0.3 (LS IV, *p* < 0.001) mg of nitrogen (N) in 100 mL of sample. Similarly, the lowest irritating properties were observed for LS IV addition (Figure 1A,B).

In order to determine the influence of the sample on the pH and the Zein parameter, a ranking transformation was performed for the non-parametric factor ANOVA/ART/.

The black points in the figures correspond to the measurement points. The data is presented as a boxplot. If two experiments are connected with a colored rectangle, such as LS I and LS IIa, there are 4 asterisks above the rectangle, which means that there is a statistically significant difference between these measurements with *p* < 0.0001.

Based on the obtained results, it can be concluded that the addition of Evening PrimroseamidopropylSulfobetaine to handwashing liquids leads to a significant improvement in the safety of using this type of preparation

### 2.2. Ability to Emulsify Fatty Soils

The cleansing process will contribute to removing fat from the skin because the impurities are embedded in the skin. This position has led to the assumption that soap’s cleansing ability and ability to dissolve fats and remove them from the skin must be closely correlated. Consequently, methods for evaluating the cleansing ability of soaps are mainly based on the study of their ability to remove fat [19]. Here, we did not observe a significant difference in the ability to emulsify fatty soils by tested soaps. All tested soaps’ results amounted on average to 5.5–6.0 (Table 1).

### 2.3. AlamarBlue™ Assay

Adding new surfactants to the soaps may affect their biocompatibility with the skin cells. Therefore, here we evaluate the effect of a newly developed product on the biological response of keratinocytes−the primary cell type in the epidermis.

**Figure 2:**
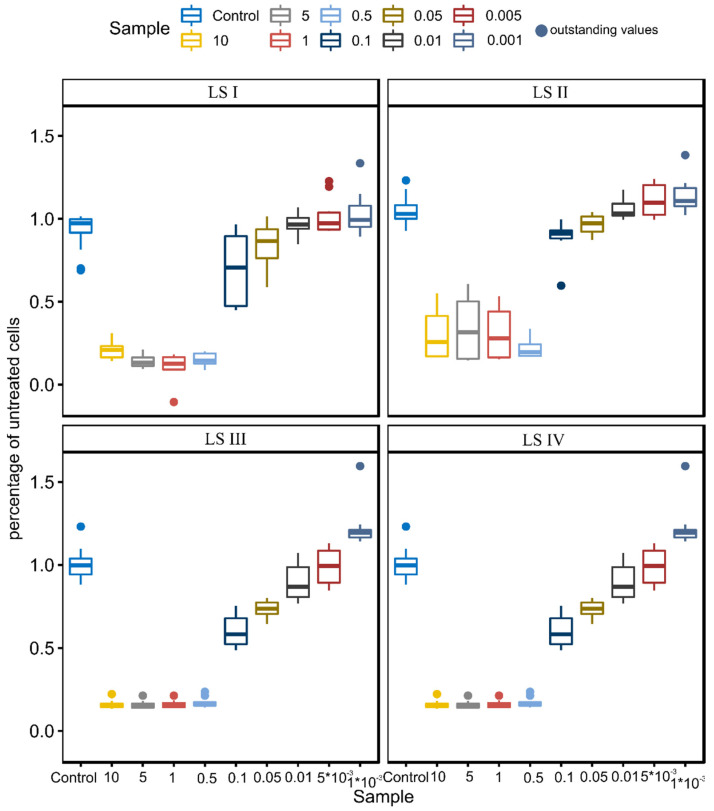
AlamarBlue™ assay of tested soaps ((LS I−base; in following soaps addition of 4% (*w/w*) surfactants: LS II−cocamidopropyl betaine; LS III−addition of cocamidopropyl hydroxysultaine; LS IV−addition of evening primroseamidopropyl sulfobetaine). Results were presented as median with quartiles (25% and 75%)., n = 10, three independent experiments.

LS I treatment caused a significant reduction of HaCaT cells counted in the AlamarBlue™ assay in a range of 0.5–10% of soap. From 0.1% to 0.001% of LS I, cells did not react (slight differences of about 5–7%). Generally, the addition of LS II and LS III did not change cell number compared to LS I. LS IV treatment significantly decreased the number of HaCaT cells in higher concentrations (0.1–40%, *p* < 0.001 and 0.05, 27%, *p* < 0.001). Interestingly, the lowest concentration of all soaps with surfactants significantly increased the HaCat cell number, with the highest differences noted for LS IV, approximately 25%, *p* = 0.002 (Figure 2).

### 2.4. Cell Viability Assay

**Figure 3:**
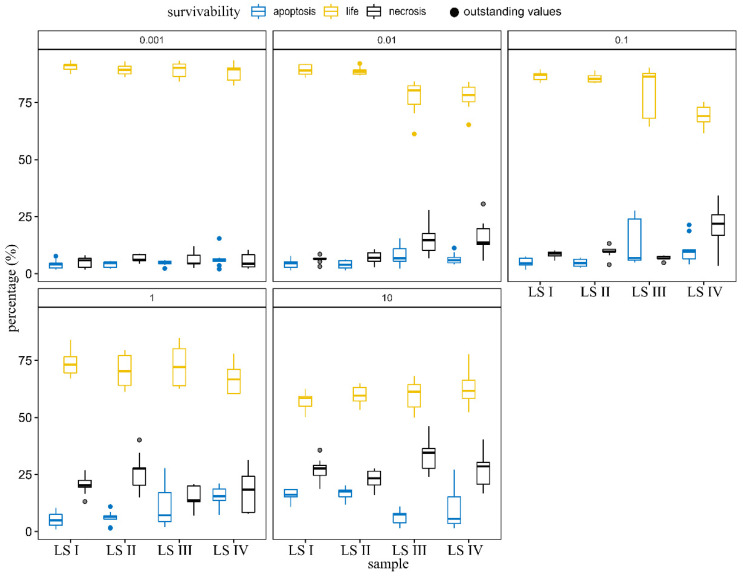
Cell viability assay of tested soaps (LS I—base; in following soaps addition of 4% (*w/w*) surfactants: LS II—cocamidopropyl betaine; LS III—addition of cocamidopropyl hydroxysultaine; LS IV—addition of evening primroseamidopropyl sulfobetaine). Results were presentedas median with quartiles (25% and 75%), n = 9, three independent experiments.

After detecting a decreased number of cells in a cytotoxic assay, we decided to evaluate the type of cell death using annexin V and propidium iodide assay, which allowed us to determine viable cells (annexin V−; PI−), apoptotic cells (annexin V+; PI−/+), or necrotic cells (annexin V−; PI+). Results from the AlamarBlue™ assay were reflected in the viability of the cells. HaCaT cells treated with 10 and 1% of LS I soap exhibited a significant reduction in cell viability (60% and 75%, respectively, *p* < 0.001), which was not visible at the lower concentrations. In the highest concentrations o LS I, the primary cell death type was necrosis. We also observed a higher apoptotic percentage of cells treated with 10 and 1% of LS I soap than untreated cells (Figure 3). Below 1%, LS I did not affect the viability of HaCaT cells. Generally, LS II treatment did not differ significantly from LS-I results. LS III and LS IV in the highest concentration (10 and 1%) did not change percentages of viable or dead cells compared to LS-I but significantly increased apoptosis at 1% (*p* < 0.001%). Although, the addition of LS III and LS IV at 0.1% (*p* < 0.001) and 0.01% (only for LS IV) significantly decreased the percentage of viable cells and increased necrosis. Moreover, LS IV significantly increased apoptosis level at 0.1% of addition in HaCaT cells. Described results are presented in Figure 3.

### 2.5. Cell Cycle Assay

**Figure 4:**
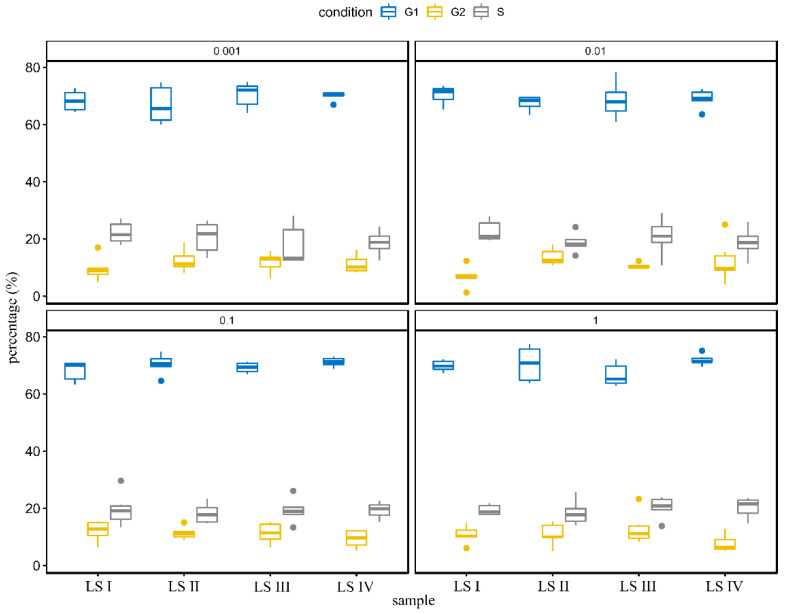
Cell cycle assay of tested soaps (LS I−base; in following soaps addition of 4% (*w/w*) surfactants: LS II−cocamidopropyl betaine; LS III−addition of cocamidopropyl hydroxysultaine; LS IV−addition of evening primroseamidopropyl sulfobetaine). Results were presented as median with quartiles (25% and 75%), n = 6, three independent experiments.

Proper cell replacement in the epidermis requires increased proliferation of keratinocytes. In the proliferation process, three main stages may be featured: (1) the resting phase of cells (G0/G1 phase), in which cells are metabolically inactive (silenced), (2) the S phase, during which the cells prepare metabolically for cell division and (3) G2 phase- the phase directly preceding mitosis [20]. We found slight but significant changes in the percentages of the cell cycle phase in all tested soaps and their concentrations. After treatment of LS I, a significantly lower percentage of the G2 phase of the cell cycle was found (in 1%, 0.01%, and 0.001%, differences up to 5%). The addition of LS III seems to slightly increase the percentage of cells in the S phase of the cell cycle (1% and 0.1%, differences up to 2%). We also found a significantly higher percentage of the G1 cell cycle phase (in 1%, 0.01%, and 0.001%, differences up to 3%). However, in our opinion, the observed differences should not significantly affect the number of cells due to minimal variation. Results are presented in Figure 4.

## 3. Discussion

Soaps for daily care should meet many different washing properties and be safe for the skin. For this purpose, surfactants are added to the soaps. Unfortunately, some anionic surfactants exhibit a negative effect on the skin. For example, it was proven that sodium salt of oxyethylated lauryl alcohol sulfate (SLES) or sodium salt of lauryl alcohol sulfate (SLS)increased the transepidermal water loss of the stratum corneum and increased dermal irritation [21]. In addition, both surfactants, in the form of monomers or micelles, can adversely affect the structural proteins of the stratum corneum. Therefore, there is a continuous search for ways to reduce the irritating effect caused by anionic surfactants. One of them is the use of auxiliary (amphoteric) surfactants. This article aimed to develop skin-friendly liquid soap prototypes using the newly synthesized amphoteric surfactant Evening Primro-seaamidopropylSulfobetaine. The authors also assessed the influence of amphoteric surfactants (and Evening PrimroseamidopropylSulfobetaine) on the safety of using this type of preparation for the skin.

Here, we found that adding Evening PrimroseamidopropylSulfobetaine to handwashing liquids significantly improves the safety of using this type of preparation. The obtained results follow the results presented in the literature. For example, Klimaszewska et al. in their work [22] proved that the addition of sulfobetaine-N-dodecyl-N-(propylpiperidinium-3-sulfonate) to liquid soaps resulted in a significant improvement of the safety of liquid soaps. The zein number values were reduced by about 16–19% compared to the reference sample. In other work by Zięba et al. [23], it was found that the replacement of cocoamdopropylbetaine, popularly used in the cosmetic industry, with new synthesized zwitterionic compounds in hair shampoo formulations, leads to products with significantly lower zein number values and a smaller increase in the pH of bovine albumin solution. This resulted in hair shampoos with increased safety for use against the skin compared to the reference shampoo. Similar results were observed by Blake [24] and Paye [25]. Furthermore, Sodium Dodecylsulfonate (SDS) solutions also significantly decrease their irritant effects following the introduction of amphoteric surfactants [26]. According to the authors of these publications, the appearance of amphoteric surfactant molecules in the system (SDS water solution) results in the formation of mixed micelles of much larger size and stability than in the case of the SDS solution.

Micelles formed in solution by the same anionic surfactant are characterized by a small size, which may result in their penetration through the skin barrier and affect the structural elements of the epidermis. Strong electrostatic repulsion of hydrophilic fragments in the molecules that build the micelle reduces its stability and leads to its fairly rapid decomposition into monomers. The addition of amphoteric surfactants increases the micelles’ size, but the system’s pH influences their stability. In an acidic environment, the amphoteric ZPC has a positive charge, it is incorporated into the micelles formed in the system, and mixed micelles are formed. These types of aggregates containing both types of surfactants are much larger in size. As a result, mutual repulsion forces are reduced, which further stabilizes the micelles. In an alkaline environment, the operation is similar to the incorporation of an anionic surfactant. Due to the difference in the structure of the hydrophilic and hydrophobic parts, the distance between the hydrophilic parts increases, which in turn translates into greater stability of the micelles. The stability of micelles in this type of system (a mixture of two different anionic surfactants) is also influenced by the difference in the size of the hydrophobic part of anionic surfactants—the greater the difference in the length of the hydrocarbon chain, the stronger the stabilization of the micelles [27].

Consequently, there is a decrease in free anionic surfactant monomers in the solution, which translates into a reduction in the possibility of binding of these surfactant molecules with protein molecules and significantly reduces the irritating effect of this type of solution. These may also explain obtained results, where irritating properties of new surfactants added to the soap, measured by bovine albumin and zein assays, were significantly decreased compared to base (LS-I).

The washing process involves removing the outer layer of grease from the surface of the skin where dirt and debris are embedded. Wolf et al. [28] presented the washing process as a complex physicochemical phenomenon in which the following steps can be distinguished: weakening of the keratin-fat binding forces due to the reduction of surface tension between water and water-resistant fat. Then, water and surfactant molecules may penetrate the finest wrinkles of the skin. In this way, the adhesion of the substrate is weakened, which is also facilitated by mechanical rubbing. The next step is transferring the fat phase to the aqueous medium. Micelles, which are formed during the emulsification of the dirt, have a negatively charged surface and are rejected by the negative charge of keratin on the skin’s surface, which significantly facilitates the given process. The last stage of the washing process concerns the dispersion/suspension of the fats particles layer with the dirt in the foam and preventing their re-deposition on the skin surface. Therefore, the ability to remove grease soiling from the skin should be optimal to provide an adequate washing effect and not affect the protective layer of the skin’s lipid film. The effect of the ability of detergents to remove fatty soils on the skin’s condition was noticed about 50 years ago in a study by Blank [29]. The author stated, “in causing inflammation, the cleanser must diffuse through the stratum corneum.”

Furthermore, Wilhelm et al., in their work, based on the results of in vivo studies, concluded that a direct interaction between surfactants and keratin proteins is responsible for the onset of skin barrier damage during the washing process. This interaction probably involves the denaturation of α-helical keratin by unfolding a coiled polypeptide protein chain [30,31]. Thus, the high ability of a cosmetic product to emulsify fats may contribute to the removal of valuable, naturally occurring hydrophobic components from the skin surface and thus disrupt the structure of the intercellular cement of the epidermis [28,32]. This may consequently lead to excessive skin dryness and irritation.

Based on the conducted study, it can be concluded that the type of applied amphoteric surfactant does not significantly affect the functionality of liquid soaps. However, it should be noted that even a small decrease in the ability to emulsify fatty dirt can bring some benefits. It may be related to the safety of this type of product in terms of its effect on the skin, namely the possibility of reducing the leaching of natural lipids present in the epidermis. The obtained results correlate with the statement that liquid soaps, compared to bar soaps, due to the pH of the product, are the most recommended for the sensitive skin of newborns and infants to guarantee the safety of the skin barrier [1]. Moreover, converging trends with the obtained results have been presented in the literature. For example, work by Sewerynet al. [33] found that the use of phosphorus derivatives of alkyl polyglucosides in the developed mild and environmentally friendly body wash cosmetics significantly reduces their ability to emulsify fats. Consequently, the drying effect on the skin caused by the washing process is significantly reduced.

In developing new ingredients for products that will have contact with human skin, cytotoxic potential should always be considered. This also applies to the surfactants synthesized in the present work. Generally, dermal soaps have contact with keratinocytes in the epidermis and do not penetrate deeper parts of the skin [19]. Furthermore, this contact is time-limited and applies to an average of 5–10 min. Therefore, ideal models for evaluating biological properties of new liquid soap products should be human or animal skin; however, due to law legislation (i.e., Directive 2010/63/EU) the number of studies performed on animals has to be limited in Europe [34]. Therefore, most studies are on cells cultured in vitro. Here we used HaCaT cells (immortalized human keratinocytes) cultured as monolayers. This research layout is more sensitive to cytotoxic substances compared to 3D differentiated keratinocytes (i.e., in EpiSkin model) or 3D multi-cell culture skin equivalent [35]. 48 h treatment of HaCat cells of LS I caused significant cytotoxicity in a range above 0.5%. This is in line with previous research in which a high concentration of soaps or other care products significantly reduces cell number after a short [36,37] and long exposition [38]. Both Cocamidopropyl Betaine (LS II) and CocamidopropylHydroxysultaine (LS III) did not affect the cytotoxic properties of LS I. The results are in agreement with other studies in which lower concentrations of Cocamidopropyl Betaine did not affect cell viability [39]. On the contrary, adding LS IV significantly increased the cytotoxicity of HaCat cells in up to 10-fold lower concentrations than LS I and increased the HaCat number in the lowest tested concentration. To the best of our knowledge, this is the first time that cytotoxicity was evaluated in soaps containing CocamidopropylHydroxysultaine and Evening PrimroseamidopropylSulfobetain.

The next step of the research was to search for a potential mechanism of decreased cell number after HaCaT liquid soap treatment. We analyzed two potential mechanisms: induction of cell death (apoptosis or necrosis) and disturbances in the cell cycle. Our results revealed only slight changes in the cell cycle phases among the studied group, which in our opinion did not impact the cell number. We found, however, some changes in the number of apoptotic and necrotic cells. Generally, in the highest cytotoxic concentrations of LS I-IV (10 and 1%), necrosis dominated. LS I and LS II caused less viability loss in HaCaT cells than LS III and LS IV. The knowledge about surfactants studied here on cell viability is limited. The effect of Cocamidopropyl Betaine in toothpaste was examined several times, concluding that the surfactant did not attenuate cell viability [39,40].

Based on the HaCaT cells analysis, it might seem that LS III and LS IV could be potentially more dangerous to epidermal cells than LS I or LS II. However, it should be noted that cells were continuously exposed to liquid soaps for a long time, which is not natural for this type of product. Moreover, soaps react with differentiated, mostly dead keratinocytes., so the cytotoxic effects of LS III and LS IV after dermal use on human skin may be unnoticeable. This may be supported by the study called Safety Assessment of Alkyl Sultaines as Used in Cosmetics from 2017, in which the biocompatibility of CocamidopropylHydroxysultaine was evaluated [41]. In this report, 41% solution of LS III caused slight erythema without edema in one of three tested rabbits after 72 h application. Moreover, 2.5% solution in humans did not irritate following a single application of the test material for 24 h during the induction. However, it irritated after repeated applications. Taken together, LS II-IV significantly improved zein number and results of the bovine albumin test. However, it may have a negative impact on keratinocytes (both increase in the following order: LS II, III, IV). Therefore, before dermal application in humans, it would be necessary to check the irritation potential following OECD guidelines for the testing of chemicals, i.e., In Vitro Skin Irritation: Reconstructed Human Epidermis Test Method [42].

## 4. Materials and Methods

### 4.1. Chemicals

As ingredients of liquids soaps following materials were used:

- Sodium Laureth Sulfate (Texapon N70, active mater 70%; BASF, Ludwigshafen, Germany);
- Cocamidopropyl Betaine (Dehyton K, active mater 30%; BASF, Ludwigshafen, Germany);
- CocamidopropylHydroxysultaine (Crodateric CAS 50LQ, active mater 50%; CRODA, Krakow, Poland);
- Evening PrimroseamidopropylSulfobetaine (sulfobetaine obtained in Department of Technology and Instrumental Analysis, Institute of Quality Science, Poznan University of Economics and Business);
- Cocamide DEA (Rokamid KAD from Rokita S.A., active matter: 98%, Brzeg Dolny, Poland);
- Urea from PPH Standard Poland;
- SodiumChloride (NaCl; POCH, Gliwice, Poland);
- Sodium Benzoate and Potassium Sorbate, KEM BS from Pol Nil S.A., Warsaw, Poland;
- Distilled water.

### 4.2. Soap Composition

The base (LS I) for the soap production with new ingredients consists of (water, sodium laureth, sulfate cocamide DEA, urea, sodium benzoate and potassium sorbate, and sodium chloride) in the proportion described in Table 2. Our new product was composed on the base and addition of 4% (*w/w*) cocamidopropyl betaine (LS II), cocamidopropylhydroxysultaine (LS III), and evening primroseamidopropylsulfobetaine (LS IV) (Table 2). In addition to LS I (bases), 4% (*w/w*) of water was added to maintain the appropriate proportions of compounds used to produce soaps.

### 4.3. Evening Primrose Oil Preparation

**Figure 5:**
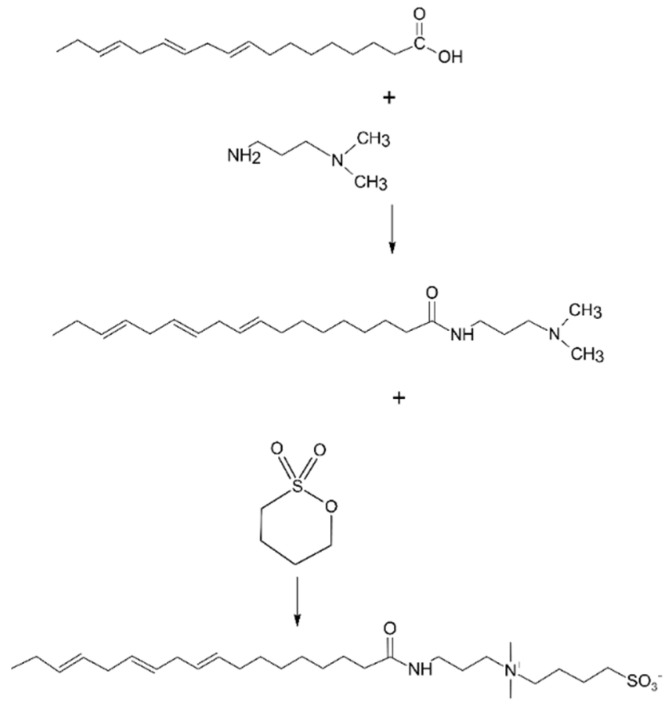
Synthesis route of Evening Primroseamidopropyl Sulfobetaine: 1H NMR (D2O) σ = 0.859 (3H, CH3), 1.218−1.286 (18H, CH2), 1.503(4H, CH2), 1.890 (4H, CH2), 2.188 (2H, CH2), 2.872 (6H, (CH3)2N+), 3.071 (4H, CH2N), 3.433 (2H, CH2S03−), 4.766 (6H, (CH). 13C NMR (D2O) σ = 15.53, 17.36, 18.47, 18,54, 18.59, 18.82, 19.09, 19,25, 19,37, 19,57, 19.64, 19.91, 20.23, 22.28, 33.86, 34.12, 41.86, 47.66, 48.47, 50.40, 125.59, 127.34, 172.16. Elemental analysis: calculated on linoleic acid derivative C 64.8%, H 10.4%, N 5.6%, S 6.4%; found C 62.0%, H 10.5%, N 5.9%, S 6.6%. IR: 3423, 2924, 2861, 1735, 1647, 1454, 1168, 1034, 891.

Evening primrose oil was purchased in one of the cosmetic ingredients stores and was classified as Evening primrose oil, cold-pressed, unrefined [www.ecospa.pl] (accessed on 9 July 2022). The synthesis procedure begins with evening primrose oil with dimethylaminopropylamine (I) reaction, conducted in an aqueous solution at a temperature of 100 ± 3 °C, under a pressure of 4 bar. As the primary Evening primrose oil fatty acid is linoleic acid, the stoichiometric amounts of Evening primrose oil (28 g) and dimethylaminopropylamine (10,2 g) were used. The reaction mixture was suspended in a small amount of water (20 mL) were placed in the pressure reactor. Then air was removed by filling the reactor with argon. The reaction mixture was mixed with a magnetic stirrer ata speed of 200 rpm for about 48 h. After 48 h, the solvent was evaporated on a pressure evaporator (Buchi). Obtained dimethylaminopropylamide (amidoamine-II) (36 g) was then reacted with 1,4-butane sultone (13 g) in ethyl acetate (50 mL). The reaction was performed in a pressure tube while the mixture was constantly stirred and heated to 70 °C. The reaction was performed for four weeks. After that, the solvent was evaporated (Buchi). Next, the mixture was extracted with anhydrous ethyl acetate. Unreacted starting materials dissolve in ethyl acetate. Finally, the product was dried in a desiccator. Schematic reactions of synthesis Evening Primrose Oil Preparation was shown of the Figure 5. 

### 4.4. Soap Production

All ingredients before addition were mixed up on a magnetic stirrer (magnetic stirrer Wigo, Poland, proximately 22 °C; time 1800 s; the rotational speed of the stirrer 400 rpm). First, an appropriate amount of water was added to a 250 mL glass flask, then surfactants (sodium laureth, sulfate cocamide DEA) and experimental additives (LS II-IV) were added. After mixing, urea was added and mixed well to obtain a homogeneous solution. Then NaCl and preservatives (sodium benzoate and potassium sorbate) were added. All was mixed for 20 min at room temperature with a magnetic stirrer. After production, soaps were stored at room temperature and protected against light exposition until analyses.

### 4.5. Irritating Properties

We performed bovine albumin and zein value assays to evaluate the potential irritating properties of new-develop soaps.

The bovine albumin test is designed to assess potential irritating properties by analysis of pH value changes caused by protein denaturation [43]. First, 50 mL of 2% BA and 50 mL of 10% appropriate liquid soap solution (both pH 5.5—adjusted by 50% of citric acid) were mixed to a glass bottle, and the initial pH was measured. Then, samples were incubated for 48 h (room temperature, in the dark), and after that pH was re-measured. Higher irritation of product is connected with the higher increase of pH value. The results were calculated from the formula: [(end pH—initial pH)/initial pH] * 100. Obtained results were presented as median with quartiles (25% and 75%), n = 7, of three independent experiments.

#### 4.5.1. The Zein Assay

The Zein assay was performed by the method described by Pezron et al. [44], with modifications. Briefly, 2 g of zein was mixed with 40 g of 10% aqueous solution of soaps in a glass bottle. Then the solution was next incubated for 1 h at 35 °C. After incubation, the amount of solubilized zein was determined by Kjeldahl nitrogen method on an automatic mineralizer (Digestor 8 AR, automatic digestor Foss, Poland) and automatic nitrogen analyzer (Kjeltec 8400, Foss, Poland). The results are presented as mg of nitrogen in 100 mL of sample (median with quartiles (25% and 75%), n = 7, three independent experiments).

#### 4.5.2. Ability to Emulsify Fatty Soils

The procedure for the ability to emulsify fatty soils was performed according to the PN-C-77003 standard. The test consisted in weighing 2 g of the liquid soap product and sudan red-colored rapeseed oil (2 g). The prepared mixture was thoroughly rubbed for about 5 min using a glass baguette. Meanwhile, tap water heated to 40 °C was prepared. The grated product was poured into a 200 cm^3^ beaker, made up to 200 cm^3^ with water, and stirred for 5 min by making half turns. After stirring the contents of the flask, it was put into the thermostat and thermostated for 30 min at 45 °C.Finally, the appearance of the emulsion was assessed using a point classification presented in Table 3.

### 4.6. Cell Culture

HaCaT cells were purchased from ThermoFisherScintific (Warsaw, Poland). The cell line is characterized as adherent, immortalized normal human keratinocytes and are widely used for the evaluation of biocompatibility of new products, in which assays are based mainly on cytotoxicity analysis [45]. We used this cell line because keratinocytes are also the first type of cells that will contact with produced soaps. The cells were grown in a standard medium: DMEM with 10% FBS and antibiotics: 50 U/mL of penicillin and 50 µg/mL of streptomycin (all from Life Technologies, Warsaw, Poland). Cells for the experiment were grown in continuous cultures. HaCaT cells were cultured in a culture bottle (25 cm^2^) and, after obtaining about 90% of confluence, were trypsinized (0.25% trypsin, 5 min., 37 °C). After trypsinization, the cells were centrifuged at 300× *g* for 5 min. Finally, the cell pellets were resuspended in 1 mL of fresh media and counted (Eve Automated Mammalian Cell Counter, Digital Bio, Seoul, Korea).

### 4.7. Cytotoxicity Assays

To evaluate the potential unhealthy effect of new-produce soaps, we used three independent cell assays: alamarBlue™ Cell Viability assay (ThermoFisher, Warsaw, Poland), viability assay (apoptosis/necrosis, Beckman Coulter, Warsaw, Poland), and cell cycle assay (propidium iodide staining, Beckman Coulter, Warsaw, Poland). Before soap addition to the cells, soaps were dissolved in the culture medium (stock 10% of soap) and filtered by microbiological filters (Corning μStar syringe filters, pore size 0.22 μm, Sigma Aldrich, Poznań, Poland).

#### 4.7.1. AlamarBlue™ Assay

AlamarBlue™ Cell Viability assay used non-toxic cell-permeable vital dye. The compound in live cells is reduced to resorufin and turns red. Colour change may be detected using absorbance at 570 and 600 nm wavelengths. Counted cells were seeded on a 96-well plate at 4 × 10^3^ cells/well. Twenty-four hours later, cells were washed twice with PBS (Life Technologies, Warsaw, Poland), and soaps dissolvent in culture media were added in growing concentration: 0.001–10% of soap. After 48 h of incubation, AlamarBlue™ was added in a fresh culture medium (1:10 Life Technologies), and absorbance (570 and 600 nm) was read on Synergy LX, Bio-Tek (Santa Clara, CA 95051, USA). Results are presented as median with quartiles (25% and 75%) percentage of control values (untreated cells), n = 10, of three independent experiments.

#### 4.7.2. Cell Viability Assay

The assay was performed according to the previously described procedure [46]. Briefly, the experiment was performed on 24-well plate. Cells (3 × 10^5^ cell/well) were seeded on the pate, and after 24 h, soaps dissolvent in culture media were added (0.001–10% of soap) for 24 h. After that, cells were harvested and resuspended in 50 μL of PBS with 4.5 μMannexin V (allophycocyanin conjugated, BD Bioscience, Warsaw, Poland) and 1 μg of propidium iodide (PI, BD Bioscience, Warsaw, Poland) and incubated (15 min., 4 °C). Then cells were analyzed by flow cytometry (CytoFlex, BecmanCulter, Warsaw, Poland, stopped after 10,000 cells). The results were obtained from Kaluza software analysis (version 2.1. Beckman Coulter, Warsaw, Poland) and presented as a percentage median with quartiles (25% and 75%) of at least three independent experiments (n = 9).

#### 4.7.3. Cell Cycle Assay

The cell cycle assay was assessed according to the procedure described elsewhere [47]. First, cells were cultured and treated with soaps as described in the cell viability procedure. After incubation with soaps (24 h), cells were trypsinized, washed twice with PBS, and incubated with 200 µL of cold 70% ethanol (4 °C) in a freezer (−20 °C) per night. Then, cells were centrifuged, washed twice with PBS, and incubated in 50 µL of the FxCycle PI/RNase Staining Solution (Thermo Fisher Scientific, Warsaw, Poland)for 30 min. Next, cells were acquired by flow cytometry (stopped after 20,000 cells). The results were analyzed with Kaluza and presented and presented as a percentage median with quartiles (25% and 75%) of at least three independent experiments (n = 6).

## Figures and Tables

**Table 1:** Ability to emulsify fatty soils of tested soaps ((LS I−base; in following soaps, addition of 4% (*w/w*) surfactants: LS II−cocamidopropyl betaine; LS III−addition of cocamidopropyl hydroxysultaine; LS IV−addition of evening primroseamidopropyl sulfobetaine). Results were presented as mean ± SD, n = 7, three independent experiments.

	Mean	SD
LS_I	6.00	0.00
LS_2	5.86	0.35
LS_3	5.71	0.45
LS_4	5.71	0.45

**Table 2:** The proportion of ingredients used for the production of new soaps.

Ingredients by International Nomenclature of Cosmetic Ingredients (INCI)	The Content of the Raw Material/the Pure Component [%*w/w*]			
	LS I	LS II	LS III	LS IV
Aqua	84.18 + 4.00	84.18	84.18	84.18
Sodium Laureth Sulfate	6.75	6.75	6.75	6.75
Cocamide DEA	2.94	2.94	2.94	2.94
Urea	1.00	1.00	1.00	1.00
Sodium Benzoate and Potassium Sorbate	0.50	0.50	0.50	0.50
Sodium Chloride	0.63	0.63	0.63	0.63
Cocamidopropyl Betaine	-	4.00		-
Cocamidopropyl Hydroxysultaine	-	-	4.00	-
Evening Primroseamidopropyl Sulfobetaine	-	-		4.00

**Table 3:** Point classification of emulsions.

Score Scale	Changes in Emulsion Appearance
0	Separated drops or a layer of clear oil
1	Rim of emulsified oil over 5 mm
2	Rim of emulsified oil from 3 to 5 mm
3	Rim of emulsified oil from 1 to 5 mm
4	Rim of emulsified oil less than 1 mm
5	The rim of the emulsified oil is very faintly visible or the emulsion is not homogenous
6	No visible rim, homogeneous emulsion

## Data Availability

The data presented in this study are available on request from the corresponding author. The data are not publicly available due to founding agreement limitations.

## Sample Availability

Samples of the compounds LS I–IV are available from the authors.
